# Abdominal Pain-Predominant Functional Gastrointestinal Disorders in Jordanian School Children

**DOI:** 10.14740/gr627w

**Published:** 2014-12-27

**Authors:** Eyad M. Altamimi, Mohammad H. Al-Safadi

**Affiliations:** a Faculty of Medicine, Mutah University, Alkarak, Jordan; b Qatar Red Crescent, Doha, Qatar

**Keywords:** Abdominal pain, School children, Functional gastrointestinal disorders, Rome III criteria

## Abstract

**Background:**

Recurrent abdominal pain (RAP) is a common complaint in children. Significant portion of them are of functional origin. This study aimed to assess the prevalence of abdominal pain-predominant functional gastrointestinal disorder (FGID) and its types in Jordanian school children.

**Methods:**

This is a school-based survey at south Jordan. Information using the self-reporting form of the Questionnaire on Pediatric Gastrointestinal Symptoms-Rome III Version (QPGS-RIII) - the official Arabic translation - was collected. Classes from academic years (grades) 6 - 8 were selected. SPSS Statistical Package Version 17 (IBM, Armonk, NY, USA) was used. Categorical data were analyzed using Fisher’s exact test, and continuous data were analyzed using *t*-test. P < 0.05 was considered significant.

**Results:**

Five hundred questionnaires were distributed, and 454 returned answered (91%). Two hundred twenty-nine (50.8%) were males. The average age of participants was 12.7 years (11 - 15 years). One hundred sixteen (25.7%) had abdominal pain-predominant FGID. Seventy-nine (68%) of them were females. Forty-seven (10.6%) had irritable bowel syndrome (IBS). Thirty-six (8%), 17 (3.8%), 11 (2.4%) and five (1.1%) had abdominal migraine, functional abdominal pain, functional abdominal pain syndrome and functional dyspepsia, respectively.

**Conclusion:**

Abdominal pain-predominant FGID has become a major health issue in Jordanian children. One of four children between the ages of 11 and 15 years exhibits at least one abdominal pain-predominant FGID. The most common form of abdominal pain-predominant FGID in our children was IBS. Females are affected more often than males. Intestinal and extra-intestinal symptoms are seen regularly with abdominal pain-predominant FGIDs.

## Introduction

Chronic pain is very common in the pediatric population. It represents both an individual and a public health issue [1]. Chronic pain adversely impacts a child’s quality of life, resulting in significantly worse physical, psychological, and social functioning, lower satisfaction with life, and poorer self-perceived health status [2-4]. Chronic recurrent abdominal pain (RAP) is one of the most frequent pain syndromes in children [5].

Apley and Naish defined RAP as a pain syndrome consisting of at least three episodes of abdominal pain over a period of not less than 3 months and severe enough to affect activities [6]. The majority of children experiencing this type of pain had no identifiable organic causes (functional disorder) [7].

Functional gastrointestinal disorders (FGIDs) are defined as variable combinations of chronic or recurrent gastrointestinal symptoms not explained by structural or biochemical abnormalities. These disorders are subcategorized by Rome criteria based on the symptoms present. Rome III criteria classify FGIDs associated with abdominal pain into the subtypes: functional dyspepsia, irritable bowel syndrome (IBS), abdominal migraine, functional abdominal pain, and functional abdominal pain syndrome [8]. The differences in pathophysiology, clinical presentation, and therapeutic interventions mandate appropriate classification of these disorders [9].

RAP has a consistently reported prevalence of 10-20% in school-age children and adolescents [10].

Office data and hospital-based studies tend to underestimate the burden of this condition, as a significant proportion of these children do not seek medical help. Epidemiological studies are needed to identify the true scope of the problem and accumulate precise data for health planning. This is particularly important because we know that, although of a benign nature, FGIDs are associated with significant negative effects on patient quality of life, as children with a continuum of issues pass into adulthood [2-4].

The Rome criteria are a valuable diagnostic tool. Applying the more inclusive Rome III criteria allowed for the classification of 87% of patients with medically unexplained chronic abdominal pain [11].

The symptom-based questionnaires depend on patient understanding of the questions and terms, which mandates that the questionnaires be in the patient’s language. Until 2012, there was no Arabic version of these questionnaires, depriving Arabic-speaking children and their treating physicians of this valuable diagnostic tool. Since a validated translated Arabic version of the questionnaires became available, the door has opened for assessing the problem in Arabic-speaking communities [12].

Rome criteria are symptom-based consensus. Pain characteristics (continuity, frequency, duration, and relation to physiological activities, such as bowel movement and eating), associated symptoms (intestinal and extra-intestinal), and impairment of daily activities form the basis of diagnosis [8].

Although FGIDs have been extensively studied, the exact etiology is not fully understood. A number of theories have been put forth, including autonomic nervous system instability [13], visceral hyperalgesia [14, 15], intestinal dysmotility [16], and stressful life events [17], none of which alone can explain the condition.

The objective of this study was to determine the prevalence of different types of abdominal pain- predominant FGID in Jordanian children and to assess the differences in pain characteristics and intestinal and extra-intestinal symptoms associated with abdominal pain-predominant FGID.

## Patients and Methods

This was a school-based survey conducted in Alkarak Governorate. Schools were selected randomly from throughout the whole geographic area and contacted in advance to obtain required permissions. Six schools participated and, in each school, classes from academic years (grades) 6 - 8 were selected. All students in selected classes who were present on the day of the survey were included.

Information regarding abdominal pain characteristics, bowel habits, and associated symptoms was collected using the self-reporting form of the Questionnaire on Pediatric Gastrointestinal Symptoms-Rome III Version (QPGS-RIII). The questionnaire was translated and validated in cooperation with the Rome Foundation, and permission to use the questionnaire was obtained from the Rome Foundation. The questionnaire was in native language and easy to understand. The questionnaire was distributed in the class rooms in an examination setting to ensure confidentiality and privacy. Children were given unlimited time to complete the questionnaire and verification was provided by the researcher.

Children with abdominal pain were categorized into the five subtypes of abdominal pain-predominant FGID using Rome III criteria for childhood FGID [8].

### Statistical methods

SPSS Statistical Package Version 17 (IBM, Armonk, NY, USA) was used. Results are expressed as mean with range. Categorical data were analyzed using Fisher’s exact test, and continuous data were analyzed using *t*-test. P < 0.05 was considered significant.

The Ethics Committee of Medical School-Mutah University approved the study. All the information collected was kept strictly confidential.

## Results

Five hundred questionnaires were distributed. Of these, 454 (91%) were completed, and 451 were included in the analysis. Three were excluded from analysis due to insufficient data. Demographic information for 451 students whose questionnaires were included is presented in Table 1.

**Table 1:** Patient Demographics

Female (N)	222
Male (N)	229
Age, mean (range) (years)	12.7 (11 - 15)

### Prevalence of abdominal pain-predominant FGID

Of the children surveyed, 116 (25.7%) fulfilled the Rome III criteria for at least one abdominal pain-predominant FGID. Of these, 79 (68%) were female. IBS was the most common condition, followed by abdominal migraine, functional abdominal pain, functional abdominal pain syndrome, and functional dyspepsia.

Abdominal migraine was significantly more common among girls, as was the total number of cases of abdominal pain-predominant FGID (Table 2).

**Table 2:** Prevalence of Abdominal Pain Predominant-FGID According to Gender

Subtype	Male*, N (%)	Female, N (%)	Total, N (%)	P
Functional dyspepsia	1 (0.44)	4 (1.8)	5 (1.1)	0.169
Irritable bowel syndrome	18 (7.9)	29 (13.1)	47 (10.6)	0.072
Abdominal migraine	5 (2.2)	31 (14)	36 (8)	0.000
Functional abdominal pain syndrome	5 (2.2)	6 (2.7)	11 (2.4)	0.731
Functional abdominal pain	8 (3.5)	9 (4.1)	17 (3.8)	0.739
Abdominal pain-predominant FGID, total	37 (16.2)	79 (35.6)	116 (25.7)	0.000

FGID: functional gastrointestinal disorder. *Male, N = 229; female, N = 222; total, N = 451.

### Pain characteristics in children with abdominal pain-predominant FGID

Pain characteristics are shown in Table 3. Abdominal pain due to functional abdominal pain was significantly less frequent than other FGID types. Pain due to IBS was significantly more severe than that due to functional dyspepsia.

**Table 3:** Abdominal Pain Characteristics According to FGID Subtype

	FD, N (%)	IBS, N (%)	AM, N (%)	FAPS, N (%)	FAP, N (%)	Total, N (%)
Frequency						
1 - 3/month	0 (0)	0 (0)	13 (36.1)	0 (0)	14 (82.4)*	27 (23.3)
1/week	0 (0)	17 (36.2)	7 (19.4)	2 (18.2)	1 (5.9)	27 (23.3)
> 1/week	5 (100)	19 (40.4)	7 (19.4)	7 (63.6)	2 (11.8)	40 (34.5)
Daily	0 (0)	11 (23.4)^†^	0 (0)	2 (18.2)	0 (0)	13 (11.2)
Intensity						
Mild	1 (20)	7 (14.9)	8 (22.2)	1 (9.1)	6 (35.3)	23 (19.8)
Moderate	1 (20)	28 (59.6)	15 (41.7)	6 (54.5)	8 (47.1)	58 (50)
Strong	2 (40)	9 (9.1)	5 (13.9)	2 (18.2)	3 (17.6)	21 (18.1)
Severe	1 (20)	3(6.4)	1(2.8)	2 (18.2)	0 (0)	7 (6)
Duration						
< 1 h	2 (20)	11 (23.4)	4 (11.1)	4 (36.4)	4 (23.5)	25 (21.6)
1 - 2 h	0 (0)	19 (40.4)	8 (22.2)	2 (18.2)	11 (64.7)	40 (34.5)
3 - 4 h	1 (20)	6 (12.8)	7 (19.4)	1 (9.1)	0 (0)	15 (13)
Most of the day	2 (40)	8 (17)	6 (16.7)	4 (46.4)^§^	2 (11.8)	22 (20)
All the time	0 (0)	3 (6.4)	1 (2.8)	0 (0)	0 (0)	4 (3.4)
Gastrointestinal symptoms						
Nausea	0 (0)	15 (32)	10 (27.8)	1 (9.1)	4 (23.5)	30 (25.9)
Bloating	1 (20)	13 (27.7)	12 (33.3)	2 (18.2)	7 (41.2)	35 (30.2)
Fullness	2 (40)	18 (38.3)	12 (33.3)	3 (27.3)	5 (29.4)	40 (34.5)
Anorexia	3 (60)	21 (44.7)	17 (47.2)	5 (45.5)	5 (29.4)	51 (44)
Vomiting	1 (20)	18 (38.3)	17 (47.2)	4 (36.4)	1 (5.9)^‡^	41 (35.3)
Extra-intestinal manifestations						
Headache	4 (80)	38 (80.9)	29 (80.6)	8 (72.7)	6 (35.3)^||^	85 (73.3)
Insomnia	2 (40)	32 (68.1)	33 (91.7)	7 (63.6)	7 (64.7)**	81 (69.8)
Pain in arms and legs	3 (60)	31 (66)	19 (52.8)	7 (63.6)	5 (29.4)	65 (56)
Faintness or dizziness	2 (40)	29 (61.7)	15 (41.7)	7 (63.6)	5 (29.4)	58 (50)
Photophobia	2 (40)	18 (38.3)	19 (52.8)	4 (46.4)	5 (29.4)	48 (41.4)
Missing school days	2 (40)	38 (80.9)^†^^†^	15 (41.7)	6 (63.6)	6 (35.3)	67 (57.8)

FGID: functional gastrointestinal disorder; FD: functional dyspepsia, N = 5; IBS: irritable bowel syndrome, N = 47; AM: abdominal migraine, N = 36; FAPS: functional abdominal pain syndrome, N = 11; FAP: functional abdominal pain, N = 17; total, N = 116. *P < 0.05 compared to all other types; ^†^P < 0.05 compared to FAPS and AM; ^‡^P < 0.05 compared to IBS, AM, and FAPS; ^§^P < 0.05 compared to IBS, AM, and FAP; ^||^P < 0.05 compared to IBS and AM. **P < 0.05 compared to all other types. ^†^^†^P < 0.05 compared to AM, FAPS, and FAP.

Sixty-seven (57.8%) of children with abdominal pain-predominant FGID missed school days. Children with IBS lost significantly more school days than children with the other subtypes (Table 3).

### Intestinal and extra-intestinal symptoms in affected children

Of intestinal symptoms, vomiting was significantly less prevalent with functional abdominal pain than with IBS (P = 0.014), abdominal migraine (P = 0.005), and functional abdominal pain syndrome (P = 0.05).

Insomnia was significantly more prevalent in patients with abdominal migraine than in patients with other abdominal pain-predominant FGID, whereas headache was relatively less common with functional abdominal pain than with the other abdominal pain-predominant FGIDs (Table 3).

## Discussion

Assessment of the burden of abdominal pain-predominant FGID in Jordanian community is a necessity. Community-based studies provide more precise estimates for addressing such health issues. In the present epidemiological survey, we demonstrated that 25.7% of Jordanian children had at least one abdominal pain-predominant FGID. IBS was the most prevalent FGID, followed by abdominal migraine and functional abdominal pain. Female gender was associated with higher prevalence of abdominal pain-predominant FGID. IBS was associated with significantly greater loss of school days than the other types of abdominal pain-predominant FGID. Insomnia was significantly more common with abdominal migraine.

RAP is a common pediatric problem. Functional etiology is the most underlying cause [18]. Rome criteria classify FGIDs associated with abdominal pain according to pain characteristics and associated gastrointestinal and non-gastrointestinal symptoms into the subtypes: functional dyspepsia, IBS, abdominal migraine, functional abdominal pain, and functional abdominal pain syndrome. The classification is important because underlying symptomatology, clinical profile, and management differ among the subtypes [8].

Epidemiological studies are important not only to estimate the scope of the problem in this age group, but also to predict the future burden of FGID during adulthood. It has already been suggested that a significant percentage of children with RAP will grow into adult IBS patients [19].

Epidemiological studies from the western hemisphere estimate that the prevalence of RAP varies from 0.3% to 19% [18]. Studies from developing countries are scarce. One study from Sri Lanka estimated the prevalence of abdominal pain-predominant FGID to be 12.5% [20]. Our study estimated that one out of four children between the ages of 11 and 16 years meets the criteria for at least one abdominal pain-predominant FGID. Our numbers are higher than reported rates, which might be related to over-reporting by the responders, considering that our study was not coupled with clinical evaluation.

In our cohort, female predominance was seen consistently in all subtypes of abdominal pain-predominant FGID. Girls were affected at double the rate at which boys were affected (35.6% vs. 16.2%), which was statistically significant (P = 0.000). Our results are in agreement with previous reports [21, 22].

Females reported higher rates of RAP, especially around the time of puberty. Hormonal changes could explain the gender difference at pubertal ages. This theory fails to explain the gender difference seen in younger children [23], whereas other theories, such as visceral hypersensitivity, could explain female predominance in prepubertal children [14, 24].

IBS was the most prevalent abdominal pain-predominant FGID, followed by abdominal migraine and functional abdominal pain. Data from developed countries show that the prevalence of IBS ranges from 6% to 14% in children and 22.0-35.5% in adolescents [25-27].

A recent Sri Lankan survey of students determined that the prevalence of IBS in children and adolescents was 4.9% based on the Rome III criteria [21]. In our study, the prevalence of IBS in children 11 - 15 years old was 10.6%. Westernization of Jordanian diet might be responsible for the increased rate compared to the Sri Lankan children.

Abdominal migraine affects 1-4% of children and is a variant of migraine headaches [28]. Onset is seen most often between the ages of 7 and 12 years [8, 28]. In the current cohort, abdominal migraine was the second most common of all abdominal pain-predominant FGIDs, with a prevalence rate of 8%, which is almost twice the reported rate. We believe that this might be in part related to differences in diagnostic criteria used.

Abdominal migraine is known to affect female disproportionately [29]. Our study also demonstrated a female predominance. In our cohort, the ratio of affected females to males was exaggerated (14% vs. 2.2%) compared to that previously reported [30]. We do not have an explanation for this difference, and we believe further studies will be needed both to confirm these rates in our population and to establish the cause underlying the elevated rates.

In our cohort, the majority of patients described their pain as infrequent, not particularly severe, and of relatively short duration (< 2 h). This is consistent with the findings of epidemiological studies with similar diagnostic criteria [21]. Previous hospital-based studies demonstrated higher levels of severity [25], as severity of symptoms is a major determinant of whether to seek medical help. The frequency of abdominal pain in patients with functional abdominal pain was lower than in all other types of abdominal pain-predominant functional abdominal pain.

Extra-intestinal symptoms were seen consistently with all types of abdominal pain-predominant FGIDs, with headache being the most common. Dong et al reported difficulty sleeping to be more common in children with IBS. However, in our cohort, this was not the case. In fact, insomnia was significantly more prevalent with abdominal migraine than with the other subtypes of abdominal pain-predominant FGID [31].

This study also demonstrated the impact of IBS on school attendance of affected children. A significantly higher percentage of children with IBS missed school compared to those with other types of abdominal pain-predominant FGID. This is consistent with previous reports of IBS in children [32].

The reason for this is not clear, especially given that no significant increase in pain severity or prevalence of intestinal or extra-intestinal symptoms was observed in IBS patients compared to those with other types of abdominal pain-predominant FGID.

The main limitation of this study was the inability to rule out organic disorders. There was no patient interview or physical examination to exclude an organic etiology for abdominal pain. However, all the epidemiological studies cited here used the same methodology.

In conclusion, abdominal pain-predominant FGID has become a major health issue in Jordanian children. One of four children between the ages of 11 and 15 years exhibits at least one abdominal pain-predominant FGID. The most common form of abdominal pain-predominant FGID in our children was IBS (10.6%), while the least common was functional dyspepsia (1.1%). Females are affected more often than males. Intestinal and extra-intestinal symptoms are seen regularly with abdominal pain-predominant FGIDs.

## Author Note

The paper was presented at a meeting organized by NASPGHAN in Chicago in October 2013.
